# Navigating Acute Psychiatric Care: Comparative Narrative Service Mapping Across England and Germany

**DOI:** 10.1192/bjo.2026.11651

**Published:** 2026-06-30

**Authors:** Alina Dietrich, Saikat Roy, Laura Fischer

**Affiliations:** 1 West London NHS Trust, London, United Kingdom; 2 Max-Planck Institute of Psychiatry, Munich, Germany

## Abstract

**Aims:**

In England, access to acute psychiatric care is increasingly affected by limited inpatient capacity, which can lead to lengthy waits in emergency departments. Emergency departments often become the default place of care for patients in acute mental health crisis while awaiting psychiatric assessment or admission. This creates strain for clinicians delivering emergency mental health care, particularly out of hours, and can lead to uncertainty and moral distress. For patients, these friction points occur when they are most unwell, shaping their experiences of care at times of acute distress and vulnerability.

We aim to explore and compare potential routes of access into acute psychiatric care across England and Germany. We are using a narrative service-mapping approach, focusing on the patient journey and the experience of psychiatry trainees, who are often responsible for out-of-hours assessment and decision making. We are hoping to stimulate reflection, discussion and generate learning across different but comparable systems.

**Methods:**

We undertook a comparative narrative service-mapping exercise based on a hypothetical patient presenting in acute mental health crisis. Using the same clinical scenario, we mapped likely pathways through community, emergency and inpatient psychiatric settings in England and Germany. Mapping was informed by clinicians’ experience of working within these systems. We worked with middle-grade and higher trainee psychiatrists involved in delivering acute and out-of-hours care in these three countries. The exercise focuses on access routes, transitions between services and areas of system pressure. Narrative elements are used to reflect how these pathways may be experienced by both the patient and the clinician providing care.

**Results:**

Across both systems, difficulties accessing care and fragmented transitions between services, were identified as sources of distress for patients and pressure for clinicians. Emergency departments emerged as settings of prolonged waiting. Differences were noted in routes to admission, gatekeeping processes and alternatives to inpatient care. These structural features shape doctors experience, including responsibility for decision-making under pressure, risk management and exposure to moral distress.

**Conclusion:**

This comparative narrative mapping highlights how the organisation of acute psychiatric services influences patient journeys and clinician experience. By foregrounding experience and reflective clinical narratives, this work aims to support discussion about shared challenges, system pressures, and potential learning across European systems. This work reflects the perspective of clinicians, using a hypothetical scenario. It is not intended to represent all patient experiences or to provide quantitative comparison between systems.

